# Results of the Schirmer tear test performed with open and closed eyes in clinically normal horses

**DOI:** 10.1186/s13028-017-0303-2

**Published:** 2017-05-31

**Authors:** Alexandra Trbolova, Masoud Selk Ghaffari

**Affiliations:** 1 Clinic of Small Animals, University of Veterinary Medicine and Pharmacy, Kosice, Slovakia; 2 Department of Clinical Sciences, College of Veterinary Medicine, Karaj Branch, Islamic Azad University, Alborz, Iran

**Keywords:** Horse, Equine, Schirmer tear test, STT, Open and closed eyes STT

## Abstract

**Background:**

The Schirmer tear test (STT) is widely used in both human and veterinary ophthalmology. Two types of STTs have been developed: STT I and SST II. The STT I measures the basal and reflex tear production and is the most widely used STT. However, several factors influence the STT results such as the person performing the test and the location of the strip placement within the conjunctival sac. The aim of this study was to measure the basal and reflex tear production (STT I) in clinically normal horses with open versus closed eyes.

**Results:**

Forty clinically healthy horses without any ocular diseases were included. On day 1, the STT I was first performed on all the horses with the eyes open followed by an STT I with closed eyes performed 30 min later. On day 2, all horses had their eyes closed during the first STT and the eyes open during the second test performed 30 min later. The mean value of the STTs performed on open eye was significantly less than the STT performed on closed eye on both days of examination.

**Conclusion:**

This study showed a small but statistically significant difference between STT values obtained with open versus closed eyes in clinically normal horses.

## Findings

Tears play an important role in maintaining the health and normal functions of the conjunctiva and cornea and deficiency in tear production results in keratoconjunctivitis sicca (KCS) [1]. The measurement of tear production is an important diagnostic test when deficiency of the lachrymal system is suspected. The Schirmer tear test (STT) devised by Otto Schirmer a century ago is widely used in both human and veterinary ophthalmology as a basic assessment of tear production [2]. Two types of STTs have been developed: STT I and STT II. The SST I is the most commonly used STT and measures the basal and reflex tear production. The STT II on the other hand evaluates basal tear production after topical application of an anesthetic and is of predictive values in animals with corneal ulceration, which do not tolerate the STT I.

Several factors influence the STT results such as inconsistencies in the absorptive capacities of STT strips due to differences in filter papers, the person performing the test, and the location of strip placement within the conjunctival sac [3].

The results of the STT in clinically normal horses have been reviewed in several studies, but no studies have focused specifically on the results of open eye STT versus closed eye STT in horses. The aim of the present study was therefore to compare the STT values obtained with open versus closed eyes in clinically normal horses.

Forty clinically healthy horses (20 males and 20 females) without any ocular diseases were included. The horses were selected based on normal physical and ocular examinations as determined by STT I, biomicroscopy, indirect ophthalmoscopy, and fluorescein staining.

STT I values were evaluated in both eyes of all horses using a commercial STT strip of a single lot number (Schirmer-Tränentest, Vet Eickemeyer, Tuttlingen, Germany). On day 1, the STT was first performed on all the horses with the eyes open followed by an STT with closed eyes 30 min later. On day 2, all horses had the eyes closed during the first STT and the eyes open during the second test performed 30 min later.

The STT was performed by inserting a STT strip inside the lower eyelid approximately one-third of the distance from the temporal to the nasal canthi for one minute. The eyelids were kept open/closed by the use of the examiner’s fingers. All examinations were carried out in the housing area of the horses at the same time of day (between 10:00 a.m. and 12:00 noon) and all the measurements were performed by the same investigator (AT). A chemical restraint was not used.

The statistical analysis was performed by using the software package SPSS 12.0 for Microsoft windows (SPSS Inc., Chicago, IL, USA). The data were reported as mean ± standard deviation (SD).

Student’s t test was used to compare the STT values obtained on each of the days for the right and left eyes and to evaluate the STT values for open eyes versus closed eyes. A P value of <0.05 was considered as statistically significant.

The obtained STT values and corresponding P values are presented in Table 1.

**Table 1:** Values of the Schirmer tear test (mean and standard deviation in mm/min) in 40 clinically normal horses measured twice daily on two consecutive days

Test day	Eye status and no. of measurements	Eye		P value
		Left	Right	
Day 1	Open T0 (n = 40)	21.5 ± 3.1	21.5 ± 3.3	1.0
	Close T30 (n = 40)	22.3 ± 3.0	21.9 ± 3.1	0.02
	P value	<0.001	0.027	
Day 2	Close T0 (n = 40)	23.7 ± 2.8	23.7 ± 2.8	1.0
	Open T30 (n = 40)	23.0 ± 3.1	22.8 ± 2.9	0.3
	P value	0.004	<0.001	

Number of measurements per eye per day = 80

The mean value of the STTs of the left and right open eyes versus the left and right closed eyes was significantly less for both days (P < 0.001). Left and right open eye measurements were not significantly different at any of the days (P = 1.0 and 0.3 for days 1 and 2, respectively). However, when analyzing the results for closed eyes measurements, the left–right values for day 1 were significantly different (P = 0.02) while they did not differ at day 2 (P = 1.0).

The overall mean and SD for all measurements on open eyes was 22.2 ± 2.9 mm/min, while the value for closed eyes was 22.9 ± 2.8 mm/min.

The study shows that the results of a STT I depend on whether the test is done on open or closed eyes as the STT I values were significantly higher for closed eyes than for open eyes.

The values of the STT I observed in the horses were similar to those reported in other studies of normal horses. Previous studies have reported the normal STT I values in horses as 20.6 ± 6.5 [4] and 24.8 ± 4.8 mm/min [5, 6].

Our results differ from those found in humans, in which the STT I values with open eyes showed significantly higher values when compared to closed eyes. In humans, possible mechanisms for higher STT values with open eyes are influence by external factors such as temperature, evaporation, and humidity [7–9]. It is also possible that the human cornea is more sensitive than the equine cornea thus producing more tears. Also, in humans, the duration of a STT measurement is generally 5 min compared to 1 min in horses. The longer time with the eyes open with the strip inserted may increase tearing [10, 11].

The specific details of lacrimal gland innervation are poorly understood in horses. Fibers from the ophthalmic division of the trigeminal nerve, facial nerve, pterygopalatine ganglion, and sympathetic fibers from the carotid plexus have been traced to the lacrimal gland. The nictitans gland surrounds the base of the third eyelid cartilage and is innervated by parasympathetic fibers from the glossopharyngeal cranial nerve [12, 13].

It is generally believed that manipulative procedures, such as corneal or conjunctival scrapings, flushing of the lacrimal apparatus, and potentially even application of bright light to an inflamed eye may result in artificially elevated STT values. For these reasons, if the STT is to be performed, it should be done very early in an ophthalmic examination [13].

The exact reason for the mild increased tear production in horses of our study with closed eye STT I is uncertain but a possible explanation could be a reflex tearing by stimulating the trigeminal nerve via the eyelid sensation. As the STT strip itself irritates the eye and stimulates the production of reflex tears, topical anesthetic eliminates this irritation and thereby eliminates the reflex tearing. Thus, in many species, STT II values are lower than STT I values [14, 15].

Several studies have evaluated the effects of age, season, environment, sex, and time of day on tear production in horses [5, 16]. The STT values can also vary depending on inconsistencies in the absorptive capacities of the STT strips due to differences in filter papers or the individual performing the test [3].

Keratoconjunctivitis sicca in horses is considered relatively rare compared to other species and is mostly associated with facial nerve paralysis resulting from head trauma, fractures of the mandible, petrous temporal or stylohyoid bones, or equine protozoal encephalomyelitis. Other reported causes include eosinophilic granulomatous dacryoadenitis and hypothyroidism. Determining the normal levels of tear secretion in healthy horses could be of help for the accurate diagnosis of KCS in this species [13, 17–19].

In conclusion, this study shows a small, but statistically significant difference between STT I measurements in normal horses with eyes either being open or closed. Although the difference may be clinically insignificant, this information is useful in two ways. First, it implies that STT I can be measured with open or closed eyes in the horse with clinically relevant accuracy and second that the difference in STT values should be taken into consideration when evaluating horses with ocular surface disease where the method of performing the test may be of more significance.
